# 3-(7,8,13,14-Tetra­hydrodi­benzo­[*a*,*i*]phen­an­thridin-5-yl)benzene-1,2-diol

**DOI:** 10.1107/S1600536810023688

**Published:** 2010-06-23

**Authors:** N. S. Karthikeyan, G. Ramachandran, K. Sathiyanarayanan, P. Raghavaiah, R. S. Rathore

**Affiliations:** a Chemistry Division, School of Advanced Sciences, VIT University, Vellore 632014, India; b School of Chemistry, University of Hyderabad, Hyderabad 500046, India; c Bioinformatics Infrastructure Facility, Department of Biotechnology, School of Life Science, University of Hyderabad, Hyderabad 500046, India

## Abstract

In the title compound, C_27_H_21_NO_2_, the half-chair conformation of the alicyclic rings gives rise to a slightly folded structure of the central tricyclic tetra­hydrophenanthridine unit. Tandem intra­molecular O—H⋯N and O—H⋯O hydrogen bonds give rise to adjacent *S*(6) and *S*(5) rings, respectively, which dictate the conformation of the 5-aryl substituent. In the crystal structure, an inter­molecular C—H⋯O contact generates chains parallel to [101]. Short O—H⋯π and C—H⋯π contacts are also observed.

## Related literature

For the medicinal and optoelectronic applications of phenanthridine derivatives and for related structures, see: Sathiyanarayanan *et al.* (2009 ▶); Rathore *et al.* (2010*a* ▶,*b* ▶). For their synthesis, see: Sathiyanarayanan *et al.* (2009 ▶); Karthikeyan *et al.* (2009 ▶).
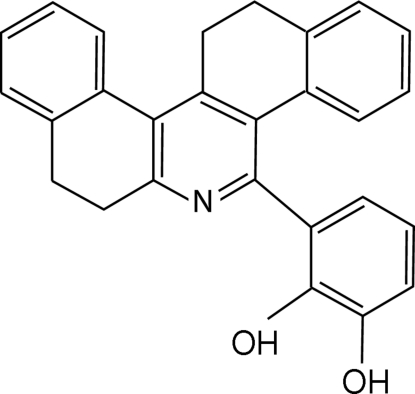


## Experimental

### 

#### Crystal data

C_27_H_21_NO_2_
*M* *_r_* = 391.45
Monoclinic, 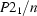

*a* = 11.4002 (10) Å
*b* = 10.2254 (7) Å
*c* = 17.3674 (16) Å
β = 106.188 (10)°
*V* = 1944.3 (3) Å^3^
*Z* = 4
Mo *K*α radiation
μ = 0.08 mm^−1^
*T* = 294 K
0.42 × 0.36 × 0.20 mm

#### Data collection

Oxford Diffraction Xcalibur Eos Gemini diffractometer
Absorption correction: multi-scan (*CrysAlis PRO*; Oxford Diffraction, 2009 ▶) *T*_min_ = 0.966, *T*_max_ = 0.983
9150 measured reflections
3974 independent reflections
2174 reflections with *I* > 2σ(*I*)
*R*_int_ = 0.036

#### Refinement

*R*[*F* ^2^ > 2σ(*F* ^2^)] = 0.042
*wR*(*F* ^2^) = 0.092
*S* = 0.86
3974 reflections
273 parameters
H-atom parameters constrained
Δρ_max_ = 0.15 e Å^−3^
Δρ_min_ = −0.19 e Å^−3^

### 

Data collection: *CrysAlis PRO* (Oxford Diffraction, 2009 ▶); cell refinement: *CrysAlis PRO*; data reduction: *CrysAlis PRO*; program(s) used to solve structure: *SHELXS97* (Sheldrick, 2008 ▶); program(s) used to refine structure: *SHELXL97* (Sheldrick, 2008 ▶); molecular graphics: *ORTEP-3* (Farrugia, 1997 ▶) and *PLATON* (Spek, 2009 ▶); software used to prepare material for publication: *SHELXL97* and *PLATON*.

## Supplementary Material

Crystal structure: contains datablocks global, I. DOI: 10.1107/S1600536810023688/bh2296sup1.cif

Structure factors: contains datablocks I. DOI: 10.1107/S1600536810023688/bh2296Isup2.hkl

Additional supplementary materials:  crystallographic information; 3D view; checkCIF report

## Figures and Tables

**Table 1:** Hydrogen-bond geometry (Å, °) *Cg*3 is the centroid of the C4–C9 ring.

*D*—H⋯*A*	*D*—H	H⋯*A*	*D*⋯*A*	*D*—H⋯*A*
O1—H1⋯N1	0.82	1.92	2.616 (2)	142
O2—H2⋯O1	0.82	2.20	2.659 (2)	115
C8—H8⋯O2^i^	0.93	2.60	3.245 (2)	127
O2—H2⋯*Cg*3^ii^	0.82	2.99	3.6649 (14)	142
C26—H26⋯*Cg*3^iii^	0.93	2.92	3.6663 (19)	139

Symmetry codes: (i) 
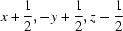
; (ii) 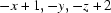; (iii) 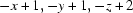.

**Fig. 1.**
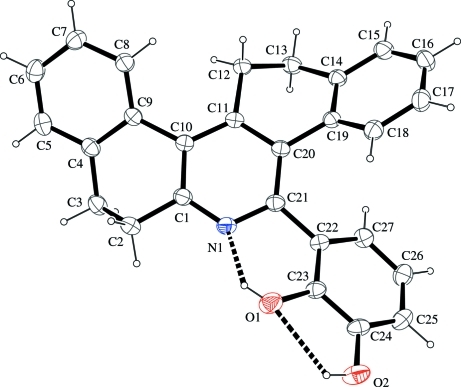
A view of (I) with non-H atoms shown as probability ellipsoids at 30% level (Farrugia, 1997). H atoms radii are on an arbitrary scale. Dashed lines indicate intra-molecular hydrogen bonds.

**Fig. 2.**
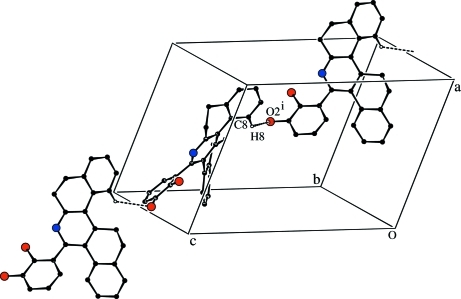
C—H···O bonded C(11) linear chain along along [1 0 1]-axis.

**Fig. 3.**
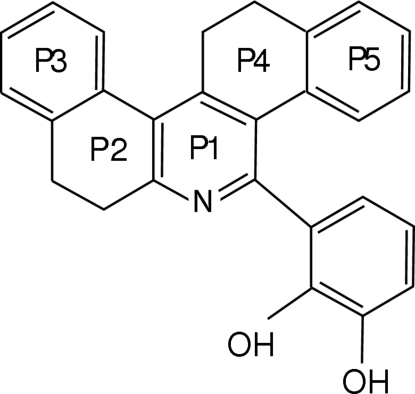
The ring nomenclature, P1—P5, adopted for (I).

## supplementary crystallographic information

### Comment

Phenanthridine derivatives are attractive candidates for medicinal and optoelectronic applications (Sathiyanarayanan *et al.*, 2009; Rathore *et al.*, 2010*a,b*). Following our new high-yielding synthetic procedure of simultaneous synthesis of phenanthridine and azabicyclo[3.3.1]nonanone, involving one-step process using tetralone and substituted benzaldehydes (Sathiyanarayanan *et al.*, 2009; Karthikeyan *et al.*, 2009), a series of one of its important pentacyclic derivative *i.e.*, 5-aryl-7,8,13,14-tetrahydro-dibenzo[a,i]phenanthridine (5ATDP) were prepared. In the present work, 5-(2,3-dihydroxyphenyl)-7,8,13,14-tetrahydro-dibenzo[a,i]phenanthridine, (I), is examined. The ring nomenclature, P1—P5 is illustrated in Supplementary Fig. 3.

The structure of (I) with adopted atom-numbering scheme is shown in Fig. 1. Alicyclic P2 (C1—C3/C4/C9/C10) and P4 (C11—C13/C14/C19/C20) rings adopt a half-chair (C2) conformation. Ring puckering parameters are as follows: ring-P2: *q*_2_ = 0.5127 (19) Å, *q*_3_ = -0.1717 (19) Å, *θ* = 108.5 (2)°, *φ* = 273.1 (2)°, and total puckering amplitude, *Q* = 0.5409 (19) Å; ring-P4: *q*_2_ = 0.5427 (18) Å, *q*_3_ = -0.1448 (18) Å, *θ* = 104.94 (18)°, *φ* = 278.09 (19)°, and total puckering amplitude, *Q* = 0.5617 (18) Å. The puckering of P2 and P4 leads to a slightly folded structure of central tetrahydro-phenanthridine tricyclic ring (N1/C1—C4/C9—C14/C19—C21), a characteristic feature among previously investigated 5ATDP analogs (Sathiyanarayanan *et al.*, 2009; Rathore *et al.*, 2010*a*).

All previously investigated 5ATDP compounds are characterized by the only plausible, cooperative C—H···N bonded *R*^2^_2_(14) closed dimers – between axial H atom of alicyclic-P4 ring and pyridine N – and these interactions are sometimes augmented by C—H···π interactions (Sathiyanarayanan *et al.*, 2009; Rathore *et al.*, 2010*a*). In contrast, the packing arrangement in (I) is different due to the presence of two strong hydroxyl donors. Two tandem intramolecular hydrogen bonds, namely O1—H1···N1 and O2—H2···O1 form hydrogen bonded two adjacent *S*(6) and *S*(5) rings (Fig. 1). The intramolecular hydrogen bonds dictate the conformation of 5-aryl substituent. An intermolecular C8—H8···O2^i^ [symmetry code (ii): 1/2 + *x*, 1/2 - *y*, -1/2 + *z*] hydrogen bond give rise to a molecular one-dimensional *C*(11) chain, parallel to [1 0 1] (Fig. 2). Two short contacts, O2—H2···.*Cg*3^ii^ [symmetry code (ii): 1 - *x*, -*y*, 2 - *z*] and C26—H26···*Cg*3^iii^ [symmetry code (iii): 1 - *x*, 1 - *y*, 2 - *z*] are also observed in the crystal structure (*Cg*3 is the centroids of (C4—C9) ring).

### Experimental

A mixture of 2-tetralone (0.01 mol) and 2,3-dihydroxy benzaldehyde (0.02 mol) was added to a warm solution of ammonium acetate (0.01 mol) in absolute ethanol (15 ml). The mixture was gently warmed on a water bath until the yellow colour changed to orange and then kept aside for overnight at room temperature. The completion of the reaction was identified with TLC. The solid obtained was separated and the crude compound was purified by silica gel column chromatography with hexane and ethyl acetate as eluant. Suitable single crystals for data collection were grown from ethanol and tetrahydrofuran mixture (in 1:1 ratio). Yield, 66°, m.p. 266–268 °C.

### Refinement

Hydrogen atoms were placed in their stereochemically expected positions and refined with the riding options. The distances with hydrogen atoms are: C(aromatic)—H = 0.93 Å, C(methylene)—H = 0.97 Å, O—H = 0.82 Å, and *U*_iso_ = 1.2 *U*_eq_(parent) or 1.5 *U*_eq_(for hydroxyl group). The torsion angles for the O—H H atoms were set with reference to a local difference Fourier map.

### Crystal data

**Table:**

C_27_H_21_NO_2_	*F*(000) = 824
*M**_r_* = 391.45	*D*_x_ = 1.337 Mg m^−^^3^
Monoclinic, *P*2_1_/*n*	Melting point: 540(2) K
Hall symbol: -P 2yn	Mo *K*α radiation, λ = 0.71073 Å
*a* = 11.4002 (10) Å	Cell parameters from 2754 reflections
*b* = 10.2254 (7) Å	θ = 2.7–28.9°
*c* = 17.3674 (16) Å	µ = 0.08 mm^−^^1^
β = 106.188 (10)°	*T* = 294 K
*V* = 1944.3 (3) Å^3^	Plate, colourless
*Z* = 4	0.42 × 0.36 × 0.20 mm

### Data collection

**Table:**

Oxford Diffraction Xcalibur Eos Gemini diffractometer	3974 independent reflections
Radiation source: Enhance (Mo) X-ray Source	2174 reflections with *I* > 2σ(*I*)
graphite	*R*_int_ = 0.036
Detector resolution: 16.3291 pixels mm^-1^	θ_max_ = 26.4°, θ_min_ = 2.7°
ω scan	*h* = −14→14
Absorption correction: multi-scan (*CrysAlis PRO*; Oxford Diffraction, 2009)	*k* = −9→12
*T*_min_ = 0.966, *T*_max_ = 0.983	*l* = −21→21
9150 measured reflections	

### Refinement

**Table:**

Refinement on *F*^2^	Primary atom site location: structure-invariant direct methods
Least-squares matrix: full	Secondary atom site location: difference Fourier map
*R*[*F*^2^ > 2σ(*F*^2^)] = 0.042	Hydrogen site location: inferred from neighbouring sites
*wR*(*F*^2^) = 0.092	H-atom parameters constrained
*S* = 0.86	*w* = 1/[σ^2^(*F*_o_^2^) + (0.0412*P*)^2^] where *P* = (*F*_o_^2^ + 2*F*_c_^2^)/3
3974 reflections	(Δ/σ)_max_ < 0.001
273 parameters	Δρ_max_ = 0.15 e Å^−^^3^
0 restraints	Δρ_min_ = −0.19 e Å^−^^3^
0 constraints	

### Special details

**Table:**

Experimental. *CrysAlis PRO*, Oxford Diffraction Ltd., Version 1.171.33.55 (release 05–01-2010 CrysAlis171. NET) Empirical absorption correction using spherical harmonics, implemented in SCALE3 ABSPACK scaling algorithm.

### Fractional atomic coordinates and isotropic or equivalent isotropic displacement parameters (Å^2^)

**Table:**

	*x*	*y*	*z*	*U*_iso_*/*U*_eq_	
C1	0.58139 (16)	0.19964 (15)	1.00791 (11)	0.0382 (4)	
C2	0.68255 (16)	0.12360 (17)	1.06347 (11)	0.0519 (5)	
H2A	0.6793	0.0332	1.0460	0.062*	
H2B	0.6730	0.1253	1.1172	0.062*	
C3	0.80504 (17)	0.18322 (18)	1.06389 (11)	0.0517 (5)	
H3A	0.8109	0.2715	1.0850	0.062*	
H3B	0.8710	0.1319	1.0978	0.062*	
C4	0.81547 (16)	0.18521 (14)	0.98005 (11)	0.0398 (4)	
C5	0.92342 (16)	0.15038 (15)	0.96280 (12)	0.0471 (5)	
H5	0.9921	0.1306	1.0046	0.057*	
C6	0.93057 (17)	0.14457 (15)	0.88518 (13)	0.0508 (5)	
H6	1.0037	0.1222	0.8748	0.061*	
C7	0.82859 (17)	0.17209 (16)	0.82289 (12)	0.0483 (5)	
H7	0.8325	0.1670	0.7702	0.058*	
C8	0.72046 (16)	0.20723 (14)	0.83870 (11)	0.0417 (4)	
H8	0.6519	0.2245	0.7962	0.050*	
C9	0.71229 (15)	0.21729 (14)	0.91707 (10)	0.0351 (4)	
C10	0.59725 (15)	0.25250 (14)	0.93686 (10)	0.0341 (4)	
C11	0.50312 (15)	0.33225 (14)	0.89077 (10)	0.0335 (4)	
C12	0.51453 (15)	0.40983 (14)	0.81910 (10)	0.0400 (4)	
H12A	0.4838	0.3582	0.7708	0.048*	
H12B	0.5999	0.4290	0.8250	0.048*	
C13	0.44307 (16)	0.53713 (15)	0.81136 (11)	0.0445 (5)	
H13A	0.4774	0.5918	0.8578	0.053*	
H13B	0.4488	0.5842	0.7640	0.053*	
C14	0.31175 (16)	0.50795 (15)	0.80502 (10)	0.0369 (4)	
C15	0.21463 (18)	0.56693 (17)	0.74949 (11)	0.0475 (5)	
H15	0.2296	0.6340	0.7173	0.057*	
C16	0.09624 (19)	0.52755 (17)	0.74136 (12)	0.0529 (5)	
H16	0.0321	0.5689	0.7043	0.063*	
C17	0.07236 (17)	0.42738 (17)	0.78777 (12)	0.0496 (5)	
H17	−0.0073	0.3987	0.7810	0.059*	
C18	0.16807 (16)	0.36996 (16)	0.84443 (11)	0.0425 (5)	
H18	0.1520	0.3028	0.8761	0.051*	
C19	0.28837 (15)	0.41046 (14)	0.85521 (10)	0.0348 (4)	
C20	0.39375 (15)	0.34712 (14)	0.91349 (10)	0.0337 (4)	
C21	0.38970 (15)	0.29631 (14)	0.98754 (10)	0.0352 (4)	
C22	0.29358 (15)	0.32283 (14)	1.02851 (10)	0.0355 (4)	
C23	0.26657 (16)	0.23013 (15)	1.07973 (10)	0.0375 (4)	
C24	0.17524 (16)	0.25250 (16)	1.11694 (10)	0.0408 (4)	
C25	0.11592 (16)	0.37071 (16)	1.10857 (11)	0.0474 (5)	
H25	0.0549	0.3856	1.1336	0.057*	
C26	0.14778 (17)	0.46731 (17)	1.06260 (12)	0.0476 (5)	
H26	0.1104	0.5488	1.0586	0.057*	
C27	0.23424 (15)	0.44406 (15)	1.02280 (11)	0.0424 (4)	
H27	0.2538	0.5099	0.9915	0.051*	
N1	0.48233 (13)	0.22200 (12)	1.03236 (8)	0.0405 (4)	
O1	0.32438 (12)	0.11211 (10)	1.09616 (8)	0.0547 (4)	
H1	0.3904	0.1158	1.0861	0.082*	
O2	0.14479 (13)	0.15665 (11)	1.16295 (8)	0.0580 (4)	
H2	0.1878	0.0922	1.1635	0.087*	

### Atomic displacement parameters (Å^2^)

**Table:**

	*U*^11^	*U*^22^	*U*^33^	*U*^12^	*U*^13^	*U*^23^
C1	0.0440 (11)	0.0378 (9)	0.0325 (10)	0.0032 (8)	0.0105 (9)	0.0031 (8)
C2	0.0597 (13)	0.0584 (11)	0.0388 (12)	0.0166 (10)	0.0157 (10)	0.0142 (10)
C3	0.0477 (13)	0.0604 (11)	0.0404 (12)	0.0116 (10)	0.0012 (10)	0.0030 (10)
C4	0.0427 (11)	0.0352 (9)	0.0386 (11)	0.0000 (8)	0.0066 (9)	0.0007 (8)
C5	0.0367 (11)	0.0421 (10)	0.0568 (14)	0.0028 (9)	0.0034 (10)	0.0006 (10)
C6	0.0399 (12)	0.0502 (11)	0.0664 (15)	0.0017 (9)	0.0215 (11)	0.0029 (11)
C7	0.0475 (12)	0.0541 (11)	0.0493 (13)	0.0027 (10)	0.0232 (11)	0.0031 (10)
C8	0.0386 (11)	0.0458 (10)	0.0413 (12)	0.0008 (9)	0.0119 (9)	0.0046 (9)
C9	0.0363 (10)	0.0319 (9)	0.0357 (11)	−0.0016 (8)	0.0078 (9)	0.0011 (8)
C10	0.0359 (10)	0.0349 (9)	0.0312 (10)	−0.0016 (8)	0.0086 (8)	−0.0001 (8)
C11	0.0374 (10)	0.0345 (9)	0.0282 (10)	−0.0033 (8)	0.0087 (8)	0.0003 (8)
C12	0.0384 (11)	0.0482 (10)	0.0355 (11)	0.0016 (8)	0.0138 (9)	0.0090 (9)
C13	0.0500 (12)	0.0440 (10)	0.0404 (11)	−0.0002 (9)	0.0143 (10)	0.0101 (9)
C14	0.0432 (11)	0.0348 (9)	0.0327 (10)	0.0029 (8)	0.0105 (9)	−0.0012 (8)
C15	0.0566 (13)	0.0459 (11)	0.0415 (12)	0.0114 (10)	0.0162 (11)	0.0074 (9)
C16	0.0505 (13)	0.0598 (12)	0.0427 (12)	0.0192 (10)	0.0036 (10)	0.0024 (10)
C17	0.0365 (11)	0.0591 (12)	0.0509 (13)	0.0032 (9)	0.0086 (10)	−0.0072 (10)
C18	0.0397 (11)	0.0449 (10)	0.0438 (12)	−0.0003 (9)	0.0131 (9)	−0.0013 (9)
C19	0.0387 (11)	0.0357 (9)	0.0304 (10)	0.0033 (8)	0.0102 (8)	−0.0022 (8)
C20	0.0368 (10)	0.0321 (9)	0.0322 (10)	−0.0031 (8)	0.0096 (8)	0.0006 (8)
C21	0.0406 (11)	0.0322 (9)	0.0339 (10)	−0.0021 (8)	0.0123 (9)	0.0016 (8)
C22	0.0401 (11)	0.0364 (9)	0.0320 (10)	−0.0022 (8)	0.0131 (8)	−0.0011 (8)
C23	0.0459 (11)	0.0356 (10)	0.0329 (10)	−0.0008 (8)	0.0142 (9)	−0.0036 (8)
C24	0.0483 (12)	0.0451 (10)	0.0309 (10)	−0.0119 (9)	0.0140 (9)	−0.0061 (8)
C25	0.0459 (12)	0.0535 (11)	0.0491 (13)	−0.0033 (10)	0.0235 (10)	−0.0110 (10)
C26	0.0479 (12)	0.0435 (10)	0.0534 (13)	0.0028 (9)	0.0171 (10)	−0.0061 (10)
C27	0.0452 (11)	0.0385 (9)	0.0442 (12)	−0.0013 (9)	0.0134 (9)	0.0003 (9)
N1	0.0468 (10)	0.0415 (8)	0.0358 (9)	0.0038 (7)	0.0160 (8)	0.0050 (7)
O1	0.0700 (10)	0.0442 (7)	0.0605 (10)	0.0078 (7)	0.0356 (8)	0.0108 (6)
O2	0.0769 (11)	0.0544 (7)	0.0543 (9)	−0.0078 (7)	0.0374 (8)	0.0032 (7)

### Geometric parameters (Å, °)

**Table:**

C1—N1	1.332 (2)	C13—H13B	0.9700
C1—C10	1.404 (2)	C14—C15	1.387 (2)
C1—C2	1.498 (2)	C14—C19	1.398 (2)
C2—C3	1.522 (2)	C15—C16	1.377 (2)
C2—H2A	0.9700	C15—H15	0.9300
C2—H2B	0.9700	C16—C17	1.377 (2)
C3—C4	1.494 (2)	C16—H16	0.9300
C3—H3A	0.9700	C17—C18	1.380 (2)
C3—H3B	0.9700	C17—H17	0.9300
C4—C5	1.392 (2)	C18—C19	1.394 (2)
C4—C9	1.404 (2)	C18—H18	0.9300
C5—C6	1.374 (3)	C19—C20	1.486 (2)
C5—H5	0.9300	C20—C21	1.400 (2)
C6—C7	1.378 (2)	C21—N1	1.357 (2)
C6—H6	0.9300	C21—C22	1.488 (2)
C7—C8	1.383 (2)	C22—C23	1.392 (2)
C7—H7	0.9300	C22—C27	1.402 (2)
C8—C9	1.394 (2)	C23—O1	1.3665 (18)
C8—H8	0.9300	C23—C24	1.389 (2)
C9—C10	1.490 (2)	C24—O2	1.3691 (19)
C10—C11	1.406 (2)	C24—C25	1.373 (2)
C11—C20	1.417 (2)	C25—C26	1.381 (2)
C11—C12	1.512 (2)	C25—H25	0.9300
C12—C13	1.522 (2)	C26—C27	1.373 (2)
C12—H12A	0.9700	C26—H26	0.9300
C12—H12B	0.9700	C27—H27	0.9300
C13—C14	1.500 (2)	O1—H1	0.8200
C13—H13A	0.9700	O2—H2	0.8200
			
N1—C1—C10	122.83 (16)	C14—C13—H13B	109.8
N1—C1—C2	116.89 (16)	C12—C13—H13B	109.8
C10—C1—C2	120.08 (16)	H13A—C13—H13B	108.2
C1—C2—C3	109.61 (14)	C15—C14—C19	119.31 (17)
C1—C2—H2A	109.7	C15—C14—C13	123.46 (16)
C3—C2—H2A	109.7	C19—C14—C13	117.12 (16)
C1—C2—H2B	109.7	C16—C15—C14	120.90 (17)
C3—C2—H2B	109.7	C16—C15—H15	119.5
H2A—C2—H2B	108.2	C14—C15—H15	119.5
C4—C3—C2	108.85 (15)	C17—C16—C15	120.36 (18)
C4—C3—H3A	109.9	C17—C16—H16	119.8
C2—C3—H3A	109.9	C15—C16—H16	119.8
C4—C3—H3B	109.9	C16—C17—C18	119.20 (18)
C2—C3—H3B	109.9	C16—C17—H17	120.4
H3A—C3—H3B	108.3	C18—C17—H17	120.4
C5—C4—C9	119.42 (17)	C17—C18—C19	121.45 (17)
C5—C4—C3	121.46 (16)	C17—C18—H18	119.3
C9—C4—C3	119.05 (16)	C19—C18—H18	119.3
C6—C5—C4	121.34 (18)	C18—C19—C14	118.65 (16)
C6—C5—H5	119.3	C18—C19—C20	122.68 (15)
C4—C5—H5	119.3	C14—C19—C20	118.49 (15)
C5—C6—C7	119.57 (17)	C21—C20—C11	118.24 (15)
C5—C6—H6	120.2	C21—C20—C19	124.05 (15)
C7—C6—H6	120.2	C11—C20—C19	117.64 (15)
C6—C7—C8	120.04 (18)	N1—C21—C20	121.02 (15)
C6—C7—H7	120.0	N1—C21—C22	112.76 (15)
C8—C7—H7	120.0	C20—C21—C22	126.08 (15)
C7—C8—C9	121.27 (17)	C23—C22—C27	117.31 (15)
C7—C8—H8	119.4	C23—C22—C21	120.33 (14)
C9—C8—H8	119.4	C27—C22—C21	122.15 (14)
C8—C9—C4	118.30 (16)	O1—C23—C24	115.39 (14)
C8—C9—C10	123.09 (16)	O1—C23—C22	123.72 (15)
C4—C9—C10	118.50 (16)	C24—C23—C22	120.88 (15)
C1—C10—C11	117.29 (15)	O2—C24—C25	119.64 (16)
C1—C10—C9	116.55 (15)	O2—C24—C23	119.91 (15)
C11—C10—C9	126.15 (15)	C25—C24—C23	120.45 (16)
C10—C11—C20	119.65 (15)	C24—C25—C26	119.33 (17)
C10—C11—C12	123.07 (15)	C24—C25—H25	120.3
C20—C11—C12	117.22 (14)	C26—C25—H25	120.3
C11—C12—C13	110.78 (13)	C27—C26—C25	120.57 (16)
C11—C12—H12A	109.5	C27—C26—H26	119.7
C13—C12—H12A	109.5	C25—C26—H26	119.7
C11—C12—H12B	109.5	C26—C27—C22	121.15 (16)
C13—C12—H12B	109.5	C26—C27—H27	119.4
H12A—C12—H12B	108.1	C22—C27—H27	119.4
C14—C13—C12	109.58 (13)	C1—N1—C21	120.44 (15)
C14—C13—H13A	109.8	C23—O1—H1	109.5
C12—C13—H13A	109.8	C24—O2—H2	109.5
			
N1—C1—C2—C3	−139.21 (16)	C17—C18—C19—C20	−177.56 (15)
C10—C1—C2—C3	35.8 (2)	C15—C14—C19—C18	3.9 (2)
C1—C2—C3—C4	−56.87 (18)	C13—C14—C19—C18	−172.52 (15)
C2—C3—C4—C5	−137.29 (16)	C15—C14—C19—C20	179.13 (15)
C2—C3—C4—C9	39.81 (19)	C13—C14—C19—C20	2.7 (2)
C9—C4—C5—C6	−1.1 (2)	C10—C11—C20—C21	8.8 (2)
C3—C4—C5—C6	175.97 (15)	C12—C11—C20—C21	−168.34 (14)
C4—C5—C6—C7	−0.8 (3)	C10—C11—C20—C19	−168.43 (14)
C5—C6—C7—C8	1.0 (3)	C12—C11—C20—C19	14.5 (2)
C6—C7—C8—C9	0.8 (2)	C18—C19—C20—C21	−34.8 (2)
C7—C8—C9—C4	−2.8 (2)	C14—C19—C20—C21	150.12 (15)
C7—C8—C9—C10	−178.89 (14)	C18—C19—C20—C11	142.20 (16)
C5—C4—C9—C8	2.9 (2)	C14—C19—C20—C11	−32.9 (2)
C3—C4—C9—C8	−174.29 (14)	C11—C20—C21—N1	−7.4 (2)
C5—C4—C9—C10	179.17 (13)	C19—C20—C21—N1	169.61 (14)
C3—C4—C9—C10	2.0 (2)	C11—C20—C21—C22	167.95 (14)
N1—C1—C10—C11	0.1 (2)	C19—C20—C21—C22	−15.1 (2)
C2—C1—C10—C11	−174.64 (14)	N1—C21—C22—C23	−32.9 (2)
N1—C1—C10—C9	−179.29 (14)	C20—C21—C22—C23	151.40 (16)
C2—C1—C10—C9	6.0 (2)	N1—C21—C22—C27	141.72 (16)
C8—C9—C10—C1	149.50 (15)	C20—C21—C22—C27	−33.9 (2)
C4—C9—C10—C1	−26.6 (2)	C27—C22—C23—O1	−174.59 (15)
C8—C9—C10—C11	−29.8 (2)	C21—C22—C23—O1	0.3 (3)
C4—C9—C10—C11	154.06 (15)	C27—C22—C23—C24	6.5 (3)
C1—C10—C11—C20	−5.2 (2)	C21—C22—C23—C24	−178.57 (15)
C9—C10—C11—C20	174.09 (14)	O1—C23—C24—O2	−3.2 (2)
C1—C10—C11—C12	171.70 (15)	C22—C23—C24—O2	175.78 (16)
C9—C10—C11—C12	−9.0 (2)	O1—C23—C24—C25	176.16 (16)
C10—C11—C12—C13	−147.04 (15)	C22—C23—C24—C25	−4.9 (3)
C20—C11—C12—C13	29.9 (2)	O2—C24—C25—C26	179.48 (17)
C11—C12—C13—C14	−57.30 (19)	C23—C24—C25—C26	0.1 (3)
C12—C13—C14—C15	−134.66 (16)	C24—C25—C26—C27	2.7 (3)
C12—C13—C14—C19	41.6 (2)	C25—C26—C27—C22	−0.9 (3)
C19—C14—C15—C16	−2.3 (3)	C23—C22—C27—C26	−3.7 (3)
C13—C14—C15—C16	173.86 (16)	C21—C22—C27—C26	−178.51 (17)
C14—C15—C16—C17	−0.8 (3)	C10—C1—N1—C21	1.4 (2)
C15—C16—C17—C18	2.2 (3)	C2—C1—N1—C21	176.29 (14)
C16—C17—C18—C19	−0.5 (3)	C20—C21—N1—C1	2.4 (2)
C17—C18—C19—C14	−2.5 (2)	C22—C21—N1—C1	−173.55 (14)

### Hydrogen-bond geometry (Å, °)

**Table:**

*Cg*3 is the centroid of C4–C9 ring.

**Table:**

*D*—H···*A*	*D*—H	H···*A*	*D*···*A*	*D*—H···*A*
O1—H1···N1	0.82	1.92	2.616 (2)	142
O2—H2···O1	0.82	2.20	2.659 (2)	115
C8—H8···O2^i^	0.93	2.60	3.245 (2)	127
O2—H2···Cg3^ii^	0.82	2.99	3.6649 (14)	142
C26—H26···Cg3^iii^	0.93	2.92	3.6663 (19)	139

Symmetry codes: (i) *x*+1/2, −*y*+1/2, *z*−1/2; (ii) −*x*+1, −*y*, −*z*+2; (iii) −*x*+1, −*y*+1, −*z*+2.
